# Treatment Experiences of Individuals With Co‐Occurring Mental Health and Substance Use Disorders and Perspectives of Mental Health Workers in Ashanti Region, Ghana

**DOI:** 10.1111/hex.70801

**Published:** 2026-08-03

**Authors:** Godfred Darko, Georgina Agyekum Anokye, Yin Morgan Yinzooya, Thomas Agengre

**Affiliations:** ^1^ Department of Public Health Education, Faculty of Environment and Health Education University of Skills Training and Entrepreneurial Development Asante Mampong Ghana; ^2^ Department of Nursing, Faculty of Nursing and Midwifery University for Development Studies Tamale Ghana

**Keywords:** co‐occurring disorders, Ghana, mental health, substance use, treatment experiences

## Abstract

**Background:**

Co‐occurring mental health and substance use disorders remain a major challenge for treatment and recovery in low‐resource settings where services are often fragmented. People with these conditions usually receive care in systems with limited coordination, financial barriers and stigma. Our study explored the treatment experiences of individuals with co‐occurring disorders and the perspectives of mental health workers.

**Methods:**

A qualitative exploratory design was used to examine the treatment experiences of service users with co‐occurring mental health and substance use disorders and the perspectives of mental health workers. Guided by an interpretivist approach and reflexive thematic analysis, 24 service users and 10 mental health workers were purposively selected. Data were collected through semi‐structured interviews between December 2025 and March 2026, audio‐recorded, transcribed verbatim and analysed using NVivo 12. The study adhered to the COREQ: a 32‐item checklist to ensure rigour, transparency and trustworthiness.

**Results:**

Five interconnected themes emerged: fragmented and unstable treatment pathways, hidden economic burden of care and relapse cycles, stigma and social withdrawal, emotional exhaustion and psychological distress and health system constraints with gaps in community follow‐up. Participants described care as discontinuous, characterised by repeated cycles of relapse and re‐engagement, financial hardship and weak coordination between services. Stigma at family and community levels discouraged timely help‐seeking. Mental health workers highlighted systemic constraints, including inadequate staffing, transport challenges and inconsistent outreach services. There was strong agreement between service users' experiences and health workers' accounts of system‐level challenges.

**Conclusion:**

Treatment experiences are shaped by interacting social, economic and health system factors that disrupt continuity of care. Strengthening integrated, person‐centred mental health and substance use services, alongside improved community follow‐up and psychosocial support, is essential to improving outcomes in similar low‐resource settings.

**Patient or Public Contribution:**

Service users with lived experience of co‐occurring mental health and substance use disorders participated in this study and contributed to the development of interview topics by sharing priority areas of concern during data collection. Their perspectives informed the interpretation of findings alongside mental health workers. There was no formal involvement of patients or the public in study design or manuscript preparation.

## 1 Introduction

Mental health and substance use disorders remain a major global public health concern [1]. They are among the leading causes of disability and premature mortality worldwide and contribute substantially to reduced quality of life and socioeconomic burden [2]. More than one in eight people globally live with a mental disorder, while substance use disorders affect hundreds of millions of individuals [3]. When these conditions occur together, they are referred to as co‐occurring disorders, a situation in which mental illness and substance use disorder exist simultaneously and interact in ways that worsen clinical outcomes [4]. Co‐occurrence is consistently associated with greater symptom severity, higher relapse rates, poorer treatment adherence and increased mortality compared to either condition alone [5].

In Sub‐Saharan Africa, the burden of co‐occurring disorders is amplified by weak health systems, limited specialist availability and poorly coordinated service delivery [6]. Although mental health and substance use conditions are often treated within the same health system, services are typically organised in parallel rather than integrated, resulting in fragmented care pathways [7, 8]. In such settings, individuals frequently move between services without continuity, contributing to delayed recovery, repeated relapse and prolonged untreated illness [9, 10].

Integrated care refers to the coordinated management of mental health, substance use and related health needs within a unified system of care [11]. However, in practice across many African health systems, including Ghana, integration remains limited [12]. Patients with co‐occurring conditions are often managed based on the most acute presenting problem, with mental health symptoms prioritised in psychiatric settings while substance use is minimally assessed as a secondary concern [13, 14]. In other instances, substance use is the focus of attention while underlying psychiatric conditions receive limited structured follow‐up [6]. This separation is reinforced by weak referral systems, limited shared care planning and inconsistent continuity across service levels [15, 16].

In Ghana, substance use among individuals receiving mental health care commonly involves alcohol, cannabis, and increasingly, opioids such as tramadol [17, 18]. These patterns are evident among young adults and reflect broader trends of early initiation and poly‐substance use in both urban and semi‐urban settings [19]. Co‐occurring disorders are frequently observed among individuals diagnosed with schizophrenia spectrum disorders, mood disorders such as depression and bipolar disorder and acute psychotic episodes complicated by substance use [20]. Facility‐based evidence consistently indicates that substance use is a common comorbid factor among psychiatric service users [21].

Mental health services are often delivered through both facility‐based and community outreach systems [22, 23]. However, care for individuals with co‐occurring disorders remains weakly coordinated, with limited integration between mental health and substance use services, inconsistent follow‐up and delayed intervention during relapse episodes [24]. These challenges are compounded by workforce shortages, uneven distribution of services and limited psychosocial support, all of which contribute to fragmented treatment pathways and recurrent hospital presentation [25, 26].

Despite the growing burden of co‐occurring mental health and substance use disorders in Ghana, most existing studies have focused on prevalence, clinical outcomes and broader health system challenges [27]. Less attention has been given to how individuals experience treatment within fragmented service systems and how these conditions shape their day‐to‐day engagement with care. Furthermore, there is limited qualitative evidence on how mental health workers navigate the challenges of managing co‐occurring disorders within routine clinical practice. Understanding both service users' experiences and the perspectives of health workers is important for identifying barriers to continuity of care and improving the delivery of integrated, person‐centred services.

### 1.1 Aim of Study

This study aimed to (1) explore the lived treatment experiences of individuals with co‐occurring mental health and substance use disorders, with particular focus on barriers, facilitators and continuity of care within fragmented service systems, and (2) examine the perspectives of mental health workers regarding the delivery of care, challenges and system‐level constraints in managing co‐occurring disorders in routine practice within the Mampong Municipality, Ashanti Region, Ghana.

## 2 Methods and Materials

### 2.1 Study Design

This study employed a qualitative exploratory design using reflexive thematic analysis to examine the treatment experiences of individuals with co‐occurring mental health and substance use disorders, alongside the perspectives of mental health workers in the Mampong Municipality, Ashanti Region of Ghana. The design was appropriate given the complex, dynamic and context‐dependent nature of co‐occurring disorders, where individual experiences of illness, treatment pathways and health system interactions are shaped by social, cultural and structural factors [28, 29]. An interpretivist philosophical orientation underpinned the study [30, 31], which enabled an in‐depth exploration of how participants constructed meaning around their treatment experiences and professional care practices. Within this interpretivist paradigm, a phenomenological approach was adopted to capture and understand participants' lived experiences of co‐occurring mental health and substance use disorders, as well as the meanings they attach to their interactions with health services and care providers. This approach was appropriate as it allowed for detailed exploration of subjective experiences from both service users and mental health workers, rather than seeking objective generalisations.

The study is reported in line with the Consolidated Criteria for Reporting Qualitative Research (COREQ) 32‐item checklist [32] (File S1).

### 2.2 Conceptual Framework Underpinning the Study

This study is guided by the socio‐ecological model [33], which helps to explain how health experiences are shaped by interacting influences at multiple levels rather than by individual factors alone. The model is useful for understanding how treatment outcomes are shaped by a combination of personal experiences, social relationships and health system conditions.

In this study, the framework was adapted to reflect the Ghanaian mental health care context. At the individual level, factors such as substance use patterns, psychological distress and relapse experiences influence engagement with care. At the interpersonal level, family support, caregiver attitudes and stigma within close relationships shape treatment continuity and help‐seeking behaviour. At the community level, broader influences such as societal stigma, unemployment and social exclusion affect recovery trajectories. At the health system level, issues such as fragmented service delivery, limited integration of mental health and substance use care, workforce shortages and inconsistent follow‐up were considered central to shaping patient pathways.

**Figure 1:**
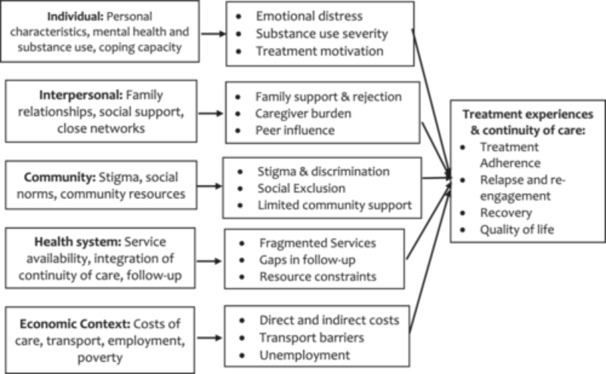
Conceptual framework adapted for the study (based on the socio‐ecological model).

Rather than serving as a rigid structure, the framework was used as a guiding lens during data collection and analysis. It helped to organise emerging findings and to interpret how different levels of influence interact to produce the observed patterns of fragmented care, relapse cycles and treatment disengagement described by participants (see Figure 1).

### 2.3 Study Setting

The study was conducted in the Mampong Municipality, a semi‐urban and rural district in the Ashanti Region of Ghana [34]. Mental health services are primarily delivered through the Mampong Municipal Hospital, which serves as the main referral centre for psychiatric and substance use‐related cases [35]. Additional primary mental health services are provided at Adidwan Health Centre and Kofiase Health Centre, which serve surrounding rural communities. Due to limited specialist capacity at these peripheral facilities, moderate to severe cases are routinely referred to the Mampong Municipal Hospital for continued management and follow‐up [36]. The mental health system operates within the Ghana Health Service decentralised framework [37], integrating facility‐based care with community outreach services. Notably, mental health nurses conduct routine monthly community follow‐up visits to support treatment adherence, psychosocial monitoring and continuity of care for patients managed in both facility and community settings. This describes the local organisation of mental health service delivery in the municipality.

### 2.4 Sampling and Participant Recruitment

A purposive sampling strategy was employed to recruit information‐rich participants with direct experience of mental health and substance use treatment services [38]. The study population comprised two groups: service users diagnosed with co‐occurring mental health and substance use disorders, and mental health professionals involved in their care. The research team had no prior therapeutic relationships with participants before recruitment.

Service users were identified through patient records from the psychiatric units of the Mampong Municipal Hospital, as well as Adidwan and Kofiase Health Centres. Eligible service users were adults with clinically documented co‐occurring mental health and substance use disorders who were currently receiving or had previously received treatment within participating facilities and were clinically stable at the time of data collection. Clinical stability was confirmed by attending mental health professionals prior to recruitment, based on absence of acute psychotic symptoms, severe mood instability and immediate relapse risk [39, 40]. This approach reduced the likelihood of interview‐related psychological decompensation.

Purposive sampling was adopted because the study aimed to generate an in‐depth understanding of lived experiences rather than produce statistically generalisable findings. In our study, participants were not selected randomly because random selection could have included individuals with limited exposure to treatment services, which would have reduced the depth and richness of the data. Instead, participants were intentionally included based on predefined clinical and experiential criteria, ensuring that each person recruited had direct and meaningful experience of co‐occurring disorders and treatment services.

Although an eligible pool of participants existed within the facilities, recruitment did not involve randomisation. All individuals who met the inclusion criteria and were willing to participate during the recruitment period were approached. This ensured transparency in selection and reduced the risk of selective inclusion by the researchers. Mental health workers included psychiatric nurses, general nurses working within mental health units, and other allied health professionals directly involved in assessment, treatment or follow‐up care.

A total of 24 service users and 10 mental health workers were recruited, giving a combined sample of 34 participants. No eligible participant declined participation in the study. The sample size was guided by the principle of data saturation. Data collection continued until thematic saturation was achieved, defined as the point at which no new codes or conceptual meanings emerged across consecutive interviews within and between participant groups. Saturation was determined through an iterative and collaborative process involving continuous comparison of emerging codes by two independent coders (G.A.A. and A.T.) and the lead investigator (G.D.) during concurrent data collection and analysis. Practically, saturation was considered achieved when three consecutive interviews within each participant group yielded no new codes or meaningful thematic variations. Saturation was achieved separately within both service user and mental health worker groups, after which data collection was terminated.

### 2.5 Data Collection Procedures and Tools

Data were collected over a 3‐month period from December 2025 to March 2026. Semi‐structured interview guides were developed based on existing literature on co‐occurring mental health and substance use disorders [41], and were adapted to the Ghanaian context through expert review and piloting to ensure cultural and contextual relevance (File S2). Separate interview guides were used for service users and mental health workers, focusing on treatment experiences, service access, adherence challenges, stigma, substance use patterns, referral pathways and perceived health system barriers and facilitators.

Interviews were conducted in either English or Twi, depending on participant preference. All Twi interviews were audio‐transcribed verbatim in Twi and subsequently translated into English by G.D. and G.A.A. of the research team. Translations were cross‐checked by a second independent researcher (A.T.), who is fluent in both languages, before coding commenced. Interviews were conducted in private and confidential settings within the health facilities after routine clinical hours to minimise disruption and ensure participant comfort and openness. Each interview lasted between 30 and 60 min and was audio‐recorded with informed consent. Field notes were also taken during interviews to capture contextual information, non‐verbal expressions and immediate reflections.

### 2.6 Ethical Consideration

Ethical approval for this study was obtained from the Ghana Health Service Ethics Review Committee (GHS‐ERC: 053/09/25). The study was conducted in accordance with the Declaration of Helsinki for research involving human participants. Written informed consent was obtained from all participants, and confidentiality, anonymity and voluntary participation were ensured. Participants were informed of their right to withdraw at any stage without consequences to their care or employment.

### 2.7 Data Analysis

Data were managed and analysed using NVivo 12 software. A reflexive thematic analysis approach, guided by Braun and Clarke's six‐phase framework, was used to examine participants' accounts. In this study, reflexive thematic analysis was understood as an interpretive process in which coding and theme development were actively shaped by the researchers' engagement with the data, rather than applied as a purely procedural technique.

Analysis began with repeated reading of transcripts to achieve familiarisation with the data. Initial codes were then generated inductively across the data set by two researchers (A.T. and G.D.). These codes were not treated as static labels but were continually refined, merged and reorganised as analysis progressed and deeper meanings emerged across interviews.

Themes were developed through an iterative process of comparing codes across and within participant groups, identifying patterns of shared meaning, and gradually clustering related codes into higher‐order concepts. This process moved back and forth between coded extracts, full transcripts and emerging thematic ideas until a coherent thematic structure was achieved.

Differences in interpretation between researchers were not treated as errors but as an expected and valuable part of reflexive thematic analysis, which is interpretive rather than consensus‐driven. These differences were resolved through discussion, critical reflection and repeated engagement with the original transcripts to ensure that final themes remained grounded in participants' accounts while acknowledging multiple possible readings of the data.

Data collection and analysis occurred concurrently, which allowed emerging insights to shape subsequent interviews and deepen conceptual development. The final thematic structure was refined through iterative review to ensure clarity, coherence and faithful representation of participants' experiences. The coding framework is provided in File S3.

### 2.8 Trustworthiness

Trustworthiness was ensured through multiple strategies. Methodological triangulation was achieved by comparing perspectives of service users and mental health workers to identify areas of convergence and divergence in treatment experiences and service delivery. Peer debriefing was conducted throughout the study among the research team to enhance reflexivity, challenge assumptions and strengthen interpretive rigour. An audit trail was systematically maintained, documenting all methodological decisions, coding processes and theme development. The research team comprised both male and female researchers. The team acknowledged their professional backgrounds in public health and mental health research within the Ghanaian health system, which shaped their engagement with the data. Reflexive practice was maintained throughout data collection and analysis through ongoing critical reflection on how these positionalities, assumptions and prior experiences could influence the interpretation of participants' accounts. These reflections were documented and discussed within the research team to support analytic transparency and minimise interpretive bias.

Although service users contributed to identifying the topics they considered important during the development of the interview guides, they were not involved in coding, theme development or interpretation of the data. These analytical stages were conducted independently by the research team using reflexive thematic analysis. Risk of interpretive bias was minimised by discussing and resolving discrepancies between participants' accounts and the researchers' emerging interpretations during team meetings. In such cases, the original transcripts were revisited to ensure that the final themes reflected participants' accounts rather than researcher assumptions. This process helped maintain a clear distinction between participant input in shaping data collection and researcher responsibility for analysis and interpretation.

## 3 Results

A total of 34 participants were included in the study, comprising 24 service users and 10 mental health workers. Among service users (Table 1), the largest proportion were aged 18–29 years (37.5%), followed by 30–39 years (29.2%), while 12.5% were aged ≥ 50 years. Most participants were male (62.5%) and single (50.0%). Educational attainment was generally low, with 75.0% having at most junior high school education. Half of the participants were engaged in informal employment (50.0%), and over one‐third were unemployed (37.5%). All service users included in the study had clinically documented co‐occurring mental health and substance use disorders and were receiving treatment or follow‐up care within participating facilities. Nearly half reported substance use for 2–5 years (45.8%).

**Table 1:** Sociodemographic characteristics of service users.

Variable	Category	*N* (%)
Age (years)	18–29	9 (37.5)
	30–39	7 (29.2)
	40–49	5 (20.8)
	≥ 50	3 (12.5)
Gender	Male	15 (62.5)
	Female	9 (37.5)
Marital status	Single	12 (50.0)
	Married	7 (29.2)
	Separated/divorced	5 (20.8)
Education level	No formal education	8 (33.3)
	Primary/JHS	10 (41.7)
	SHS or higher	6 (25.0)
Employment status	Unemployed	9 (37.5)
	Informal employment	12 (50.0)
	Formal employment	3 (12.5)
Substance use duration (years)	< 2 years	5 (20.8)
	2–5 years	11 (45.8)
	> 5 years	8 (33.3)

Among mental health workers (Table 2), half were aged 35–44 years (50.0%), and the majority were female (60.0%). Psychiatric nurses constituted the largest professional group (50.0%), followed by community psychiatric nurses (30.0%). Work experience was relatively balanced across categories, with 40.0% reporting 6–10 years of experience. Most participants worked in hospital‐based settings (60.0%), while 40.0% were involved in community outreach activities.

**Table 2:** Sociodemographic characteristics of mental health workers.

Variable	Category	*N* (%)
Age (years)	25–34	3 (30.0)
	35–44	5 (50.0)
	≥ 45	2 (20.0)
Gender	Male	4 (40.0)
	Female	6 (60.0)
Professional cadre	Psychiatric nurse	5 (50.0)
	Community psychiatric nurse	3 (30.0)
	General nurse (mental health unit)	2 (20.0)
Years of experience	1–5 years	3 (30.0)
	6–10 years	4 (40.0)
	> 10 years	3 (30.0)
Work setting	Hospital‐based	6 (60.0)
	Community outreach	4 (40.0)

## 4 Themes

Five main themes emerged from the analysis: fragmented and unstable treatment pathways, the economic burden of care, stigma and social withdrawal, emotional exhaustion and health system constraints. These findings show how a combination of personal circumstances, social influences and health system factors shape treatment experiences and continuity of care. Service user quotations are labelled as ‘SU’ followed by a participant number, while mental health workers are labelled as ‘MW’.

### 4.1 Theme 1: Fragmented and Unstable Treatment Pathways

Participants described their treatment journeys as highly unstable and non‐linear, characterised by repeated cycles of disengagement and re‐engagement with care. Rather than progressing along a clear and steady recovery pathway, treatment was experienced as fragmented and cyclical. This was also blended with periods of perceived improvement often followed by relapse, interruption of care and return to treatment. These cycles were shaped by fluctuating health status, personal circumstances and difficulty maintaining sustained engagement with services over time.

Service users described how moments of perceived recovery often led to premature disengagement from care, followed by deterioration and renewed need for treatment. SU04 explained this fluctuating trajectory:

Every time I feel I am getting better, something pulls me back. I go for treatment, I stay for some time, and then I leave because I feel okay and sometimes don't have money again. When I relapse, I come back worse than before… It feels like I am moving in circles without real progress. Even when I want to stay in treatment, life conditions push me out.

(SU04)

Beyond relapse‐related interruptions, participants also described the emotional and practical burden of repeatedly re‐entering the treatment system. SU11 emphasised the fatigue associated with this repeated engagement:

I have to repeat my story many times. It makes me tired, and sometimes I stop going. But when things become worse, I return again.

(SU11)

For many participants, this pattern created a sense of stagnation and uncertainty, where recovery felt temporary and unstable rather than cumulative. Service users often described their treatment experience as one of ‘starting again and again’, with little perceived continuity across episodes of care. This repetition contributed to frustration and a diminished sense of progress, even among those who remained motivated to continue treatment.

### 4.2 Theme 2: Hidden Economic Burden of Treatment and Relapse Cycles

Economic hardship was consistently reported across participants as a key factor shaping treatment adherence and relapse patterns. Although services are formally provided within public health facilities, indirect costs associated with transport, lost income and unstable employment emerged as significant barriers to sustained engagement in care. These financial pressures were often cyclical, intensifying during relapse episodes and recovery phases.

SU09 described persistent financial strain and dependency on informal borrowing to sustain treatment attendance:

Even when I want to continue treatment, I don't have money for transport. Sometimes I borrow before I can come to Mampong Municipal Hospital. When I get better, I try to work small, but when I relapse, everything stops again. My family is tired of supporting me, so I struggle alone most times. This period has been the most challenging times so far and I can't blame them.

(SU09)

SU15 highlighted how repeated relapse episodes disrupted vocational training and long‐term livelihood development:

I used to work as a mechanic apprentice, but when I started treatment, I stopped going regularly. Now I cannot maintain the job. Every relapse pushes me further away from learning the skill. I feel stuck because I cannot work well and I cannot also stop treatment.

(SU15)

Mental health workers corroborated these experiences, emphasising transport costs and income loss as structural barriers to continuity of care. MW06 noted:

Most of our patients cannot sustain treatment because of financial constraints. Transport alone is a major barrier, especially for those coming from rural communities. When they relapse, they also lose their jobs, which makes reintegration into care even more difficult.

(MW06)

Participants' narratives highlight that economic hardship, such as transport costs, income instability and reliance on informal financial support, were frequently described as influencing treatment engagement and continuity.

### 4.3 Theme 3: Stigma, Moral Judgement and Social Withdrawal

Stigma emerged as a pervasive influence shaping both help‐seeking behaviour and social reintegration. Participants reported stigma at multiple levels, including family rejection, community labelling and perceived judgement within health facilities. Substance use history was particularly associated with moral judgement, which reinforced self‐isolation and delayed treatment seeking.

SU02 described profound social exclusion and internalised stigma:

People in my community think I am a bad person because of drug use. Even when I try to change, they don't believe me. Some family members avoid me. When I go out, I feel like people are watching me. It makes me stay indoors most times, and when I am alone, I think too much and sometimes I go back to drugs.

(SU02)

SU18 further highlighted how anticipated stigma influenced health‐seeking delays:

Sometimes I feel ashamed to even go to the hospital. I think maybe the nurses are also judging me. So I delay going for help until I am very sick or very bad again. That delay makes my condition worse.

(SU18)

From the service delivery perspective, MW01 observed that stigma also operates at household and community levels, influencing disclosure and treatment adherence:

Families sometimes hide patients because of stigma. They do not want neighbours to know. This delays treatment and reduces follow‐up. Even when we visit homes, some relatives are not cooperative.

(MW01)

Participants' experiences reflect that stigma, which manifests as community rejection, internalised shame and anticipated judgement, significantly influenced both help‐seeking behaviours and social reintegration

### 4.4 Theme 4: Emotional Exhaustion and Psychological Distress

Participants consistently described high levels of emotional exhaustion, hopelessness and psychological instability associated with chronic relapse cycles and prolonged treatment journeys. Emotional distress was often compounded by shame, uncertainty about recovery and fear of disappointing family members.

SU07 provided a detailed account of emotional fatigue and psychological struggle:

There are days I wake up and feel like I don't want to continue anything. I feel tired of going back and forth for treatment. Sometimes I feel better, but my mind is not stable. I think about my past mistakes and I feel ashamed. I also feel like I am disappointing my family because I keep going back to the hospital. Even when I try to stay strong, something inside me breaks again and I return to drinking or using drugs.

(SU07)

SU12 similarly described emotional instability and uncertainty about recovery:

It is not easy living like this. You feel better today and worse tomorrow. Sometimes I cry alone because I don't know if I will ever fully recover. The thoughts in my mind are always heavy.

(SU12)

Mental health workers acknowledged this emotional burden, with MW09 noting that limited psychosocial support contributes to patient exhaustion:

Many patients are emotionally exhausted. They want recovery but lack continuous psychosocial support. We provide counselling, but the frequency is not enough to sustain long‐term recovery.

(MW09)

Participants' accounts indicate that prolonged treatment journeys and recurrent relapse cycles were associated with significant emotional exhaustion, psychological instability and feelings of hopelessness.

### 4.5 Theme 5: Health System Constraints and Community Follow‐Up Gaps

The final theme captures structural and operational limitations within the mental health service delivery system, particularly in relation to staffing shortages, inconsistent outreach and delayed follow‐up care. Participants indicated that while community follow‐up systems exist, they are irregular and constrained by logistical challenges.

MW04 explained these systemic limitations:

We do our best, but the workload is high. The number of patients is increasing, but staffing is limited. Community follow‐up is done monthly, but sometimes transport issues delay visits. This affects continuity of care.

(MW04)

MW07 further emphasised inconsistencies in outreach services:

We rely on patients to come to us, but many cannot. The outreach system helps, but it is not consistent. Sometimes patients relapse before we even reach them.

(MW07)

Service users confirmed these gaps in continuity. SU23 noted:

Sometimes they come to check on me at home, but it is not regular. So when I start feeling unwell again, there is no early intervention.

(SU23)

Narratives from respondents indicate that health system limitations, including staffing shortages and inconsistent community follow‐up, affected continuity of care and delayed early intervention during relapse episodes.

### 4.6 Facilitators of Treatment Engagement and Recovery

Although most narratives across the five themes focused on barriers to care and disrupted treatment pathways, participants also described important facilitating factors that supported engagement with treatment and recovery over time. These facilitators operated at the individual, interpersonal and health service levels.

At the interpersonal level, family support was repeatedly described as a key protective factor. Several service users explained that continued encouragement from relatives helped them to remain connected to care, even when adherence was inconsistent. In many cases, families played both emotional and practical roles. One participant reflected:

If not for my mother, I think I would have stopped treatment completely. Even when I go back to drinking, she still talks to me and brings me back to the hospital. She does not insult me. She just keeps telling me that I can still change.

(SU03)

Another service user described how family involvement created a sense of responsibility that motivated continued care‐seeking:

Sometimes I don't feel like going again, but when I think about my children, I force myself. My sister also checks on me regularly. That support is what keeps me going back even after many setbacks.

(SU16)

Mental health workers also confirmed that family engagement often made a noticeable difference in adherence and recovery trajectories when relatives remained involved beyond crisis periods:

Patients who have supportive families tend to return for treatment more consistently. Even when relapse happens, the family brings them back quickly. Without that support, many completely drop out.

(MW02)

At the health system level, participants emphasised the importance of therapeutic relationships with mental health workers. Trust, respectful communication and non‐judgemental attitudes were repeatedly highlighted. For many, the attitude of health workers shaped whether they felt safe returning to services:

Some nurses really understand us. They don't treat us like we are useless because of drug use. That kind of care makes it easier to come back even after disappearing for some time.

(SU20)

Mental health workers similarly reflected on the importance of maintaining openness and continuity in care relationships, even when patients defaulted:

We try not to close cases completely because many patients come back. If they feel welcomed, they re‐engage faster. But if they feel judged, they may not return at all.

(MW05)

At the individual level, participants described resilience strategies that sustained recovery efforts despite repeated setbacks. These included personal determination, spiritual coping and internal motivation to regain control over their lives. Faith in a higher power was frequently mentioned as a source of strength during difficult periods:

I always pray that God will help me stop completely. Even when I fail, I still believe I can change. That belief is what brings me back.

(SU12)

Others described an internal decision to persist despite repeated relapse cycles:

It is not easy, but I have decided not to give up on myself. Even if I go back, I still come again for treatment. One day I believe I will be free from this.

(SU09)

Mental health workers also observed that patients who demonstrated persistence in returning to care, even after long gaps, often showed gradual improvement over time:

Some patients may default many times, but if they keep returning, we see progress eventually. Recovery is not straight; it happens slowly through repeated contact.

(MW01)

### 4.7 Cross‐Perspective Triangulation of Findings

A comparison of narratives from service users and mental health workers demonstrated strong convergence across all five themes (Table 3). Both groups consistently described co‐occurring mental health and substance use disorders as conditions shaped by fragmented service delivery, economic hardship, stigma, emotional distress and weak community follow‐up systems.

**Table 3:** Cross‐perspective triangulation of findings.

Theme	Service users (SU)	Mental health workers (MW)
Fragmented treatment pathways	Repeated movement between facilities, interrupted care, loss of continuity	Poor integration of mental health and substance use services, patient ‘drop‐off’
Economic burden	Transport costs, borrowing, loss of work, inability to sustain treatment	Financial barriers and transport constraints reduce adherence and increase relapse
Stigma and social withdrawal	Community rejection, shame, delayed care‐seeking, isolation	Families conceal patients; stigma reduces follow‐up and disclosure
Emotional distress	Hopelessness, shame, psychological exhaustion linked to relapse	Limited psychosocial support; emotional fatigue observed among patients
Health system constraints	Irregular home visits, delayed intervention during relapse	Staff shortages, workload, inconsistent outreach services

Service users primarily articulated lived experiences of treatment disruption, relapse cycles and social and financial strain. Mental health workers, on the other hand, described corresponding system‐level constraints, including limited integration of services, staffing shortages, transport barriers for outreach and inadequate psychosocial support structures.

No major contradictions were identified across all domains. However, differences in emphasis were observed between service users and mental health workers. Instead, the findings reflected complementary perspectives, in which the lived experiences of service users aligned with the structural and operational explanations provided by mental health workers.

## 5 Discussion

Interpreted through a socio‐ecological lens, the findings reveal that co‐occurring mental health and substance use disorders in the Mampong Municipality are sustained within a reinforcing system of structural, social and economic constraints that continuously disrupt continuity of care. Care pathways were experienced as structurally fragmented, resulting in unstable and non‐linear treatment trajectories. These cyclical patterns reflect systemic discontinuity embedded within service organisation and weak integration between mental health and substance use services [42]. This interpretation is consistent with evidence from Ghana, where fragmentation between facility‐based and community mental health care undermines continuity and long‐term engagement [43].

This structural instability is also evident in participants' descriptions of treatment as something they repeatedly move in and out of, rather than a steady process of recovery. Care is often interrupted, resumed and interrupted again, with only short‐lived periods of stability before relapse or disengagement. Taken together, these patterns suggest that treatment operates in episodes rather than as a continuous pathway [10]. Importantly, these cycles are better understood as the outcome of gaps in service continuity and support systems, rather than as simple non‐adherence [44]. It reflects a system that struggles to hold people consistently within care long enough for recovery gains to accumulate [45].

Economic vulnerability emerged as a continuous and dynamic constraint shaping treatment engagement. Transport costs, unstable employment and reliance on informal financial networks were not merely background conditions but actively structured relapse and recovery cycles. Financial breakdown frequently preceded treatment discontinuation, while relapse further deepened economic insecurity, creating a self‐reinforcing cycle of vulnerability. Similar patterns have been documented in SSA, where indirect costs significantly limit sustained mental health service utilisation [46]. In South West Nigeria, even where services are geographically available, transport and productivity‐related costs continue to limit adherence and follow‐up [47]. These findings reinforce that economic insecurity is constitutive of illness trajectories in low‐resource settings.

Stigma emerged as a multi‐layered mechanism shaping delayed care‐seeking, treatment discontinuation and social withdrawal. Anticipated, enacted and internalised stigma operated across community, family and perceived institutional levels, indicating that moral framings of substance use extend into health system interactions [48]. This aligns with evidence from Uganda, where substance use is strongly moralised within communities, contributing to delayed help‐seeking and concealment of illness [49]. In Nigeria, anticipated stigma within both households and health services similarly limits disclosure and continuity of care [50].

Emotional distress, which includes exhaustion, hopelessness and psychological instability, should be understood as an emergent product of repeated treatment disruption rather than solely a symptom of underlying disorders [51]. The cyclical experience of relapse followed by short‐lived periods of stabilisation contributes to what can be described as stalled recovery, where individuals repeatedly re‐enter treatment without experiencing a sense of cumulative progress. Over time, this repetition creates emotional fatigue, as patients are required to recount their histories multiple times and navigate the same service pathways after each relapse. This aligns with the concept of treatment fatigue but suggests a deeper structural origin rooted in system instability [52]. Facility‐based evidence from Norway indicates that inconsistent psychosocial support contributes to sustained psychological distress among individuals with substance use disorders [53], while multi‐level evidence links fragmented addiction and mental health systems to reduced psychological resilience over time [54].

Health system constraints further reinforce these dynamics [55]. Workforce shortages, inconsistent outreach and weak follow‐up mechanisms limit timely intervention during relapse episodes, transforming care into an episodic rather than continuous process [56]. Although community mental health services exist within the Ghanaian system, their effectiveness is constrained by logistical and human resource limitations that reduce continuity of care [57]. In South Africa, irregular outreach due to resource constraints weakens early intervention capacity [58], while in Malawi similar decentralised system weaknesses contribute to inconsistent follow‐up and fragmented care pathways [59].

Despite the dominance of structural, social and economic barriers shaping care trajectories, the data indicate that recovery was intermittently supported by relational and individual resources. Family involvement occasionally facilitated re‐engagement with services after relapse, consistent with evidence from a facility‐based study in Scotland, where families often act as informal support systems in contexts of weak formal continuity of care [60]. Similarly, empathetic and non‐judgemental interactions appeared to encourage return to treatment, aligning with a study by Reyre et al. [61], which identifies therapeutic alliance as an important factor in retention within fragmented mental health systems. At the individual level, repeated attempts to re‐engage with care, alongside spiritual coping and personal determination, reflected the non‐linear nature of recovery commonly described in substance use literature [62].

Across all domains, strong convergence between service users and mental health workers reinforces the robustness of the findings. Both groups articulated a shared systemic reality characterised by fragmented care, economic precarity, stigma, emotional burden and weak follow‐up systems, albeit from different positional standpoints. This alignment strengthens interpretive validity and indicates that treatment instability is not primarily an individual‐level phenomenon but an emergent property of interacting social and health system structures.

### 5.1 Strengths and Limitations

The strengths of this study lie in three main areas. First, the inclusion of both service users and mental health workers provided a multi‐perspective understanding of co‐occurring mental health and substance use disorders, which enhanced the depth and credibility of the findings. Second, the use of reflexive thematic analysis enabled a rich and nuanced interpretation of complex treatment experiences within their broader social and structural context. Third, methodological rigour was strengthened through strategies such as triangulation, member checking, peer debriefing and the maintenance of an audit trail, all of which enhanced the trustworthiness and transparency of the study.

However, some limitations should be acknowledged. The study was conducted within a single municipality in the Ashanti Region. As such, the findings are not generalisable to all individuals with co‐occurring mental health and substance use disorders in Ghana or other settings, but rather should be understood as context‐specific insights into the experiences of the participants involved. Additionally, although efforts were made to ensure openness during interviews, participants' responses may still have been influenced by recall bias or social desirability bias given the sensitive nature of substance use and mental health experiences. Finally, the study focused primarily on service users who were clinically stable at the time of data collection, which may have excluded the perspectives of individuals in acute phases of illness who may experience even greater barriers to care.

### 5.2 Implications for Policy and Practice

Our findings for this study point to the need to rethink how care is delivered for people with co‐occurring mental health and substance use disorders in Ghana. At the practice level, care needs to move away from short, disconnected episodes of treatment toward continuous, person‐centred support. Regular and reliable follow‐up, especially through community outreach and case management, is important to reduce repeated disengagement and relapse patterns. Adding routine psychosocial support into both facility and community services can also help ease the emotional strain and treatment fatigue. Training mental health workers on co‐occurring disorders would further support earlier identification, better coordination of care and smoother follow‐up across services.

At the policy level, the study highlights the need to move beyond policy intentions toward real integration of mental health and substance use services within the health system. Even though the Mental Health Act (Act 846) provides a framework, gaps remain in staffing, referral systems and community follow‐up. Policies should therefore support stronger community mental health services with the logistics needed for outreach work to happen consistently. The cost of care also needs attention, especially transport costs that make it difficult for people to stay in treatment. Support such as transport subsidies or other social protection measures could make a real difference for vulnerable service users. Stigma also remains a major barrier, so anti‐stigma work should be built into national mental health and substance use programmes, not treated as an add‐on.

## 6 Conclusion

This study shows that care for co‐occurring mental health and substance use disorders in the Mampong Municipality is shaped by persistent system, social and economic constraints that disrupt continuity of treatment. Rather than following a stable recovery pathway, service users move through repeated cycles of disengagement, relapse and re‐entry into care, reflecting gaps in coordination across services, weak follow‐up systems and everyday financial hardship.

Both service users and mental health workers describe the same underlying reality: a fragmented system that is not designed to sustain long‐term engagement for people with complex needs. Recovery is therefore less a linear clinical process and more a negotiated outcome shaped by whether care remains accessible, continuous and socially supported.

These findings point to the need for targeted system‐level reforms. Strengthening integrated delivery of mental health and substance use services at both facility and community levels is essential through improved referral pathways. Community follow‐up should be resourced with reliable transport and staffing support to ensure timely contact during relapse risk periods. In addition, reducing indirect costs of care, especially transport expenses, through targeted subsidies and social protection mechanisms could improve sustained engagement in treatment. Finally, embedding structured anti‐stigma interventions within routine mental health services is critical to improving early help‐seeking and reducing treatment delay.

## Author Contributions

**Godfred Darko:** conceptualisation, writing – original draft, writing – review and editing; validation, methodology, formal analysis, software, supervision. **Georgina Agyekum Anokye:** conceptualisation, methodology, writing – review and editing. **Yin Morgan Yinzooya:** writing – review and editing, methodology. **Thomas Agengre:** methodology, writing – original draft.

## Funding

The authors have nothing to report.

## Ethics Statement

Ethical approval was obtained from the Ghana Health Service Ethics Review Committee (GHS‐ERC: 053/09/25). Written informed consent was obtained from all participants. The study was conducted in accordance with the Declaration of Helsinki.

## Consent

The authors have nothing to report.

## Conflicts of Interest

The authors declare no conflicts of interest.

## Use of Artificial Intelligence Tools

Artificial intelligence‐based tools (ChatGPT, OpenAI) were used to support language refinement and improve clarity during manuscript preparation. The content, analysis, interpretation of findings and final scientific conclusions were developed and verified by the authors. The authors take full responsibility for the integrity of the work.

## Supporting information


Supporting File 1

Supporting File 2

Supporting File 3

## Data Availability

The data that support the findings of this study are available on request from the corresponding author. The data are not publicly available due to privacy or ethical restrictions.
