# Supplementary material for: Treatment Experiences of Individuals With Co‐Occurring Mental Health and Substance Use Disorders and Perspectives of Mental Health Workers in Ashanti Region, Ghana
Source: Health Expect. 2026 Aug 3;29(4):e70801. doi: 10.1111/hex.70801 (PMC13430574; doi:10.1111/hex.70801)
Supplement: Supplementary file 1 — Supporting File 1 [file HEX-29-e70801-s002.docx]

**SUPPLEMENTARY FILE 1**

**COREQ 32-Item Checklist**

**Domain 1:** Research team and reflexivity

| **No.** | **Item** | **Description** | **Reported** | **Manuscript Section** | **Page No.** |
| --- | --- | --- | --- | --- | --- |
| 1 | Interviewer/facilitator | Interviews conducted by trained qualitative researchers | Yes | Data collection procedures | 8 |
| 2 | Credentials | Research team academic background stated | Yes | Study design | 9 |
| 3 | Occupation | Public health / mental health researchers | Yes | Study design | 9 |
| 4 | Gender | Not explicitly reported | Yes | Methods/  Trustworthiness | 9 |
| 5 | Experience and training | Trained in qualitative research methods | Yes | Data collection procedures | 6 |
| 6 | Relationship with participants | No prior relationship established | Yes | Sampling and recruitment | 6 |

**Domain 2: Study design**

| **No.** | **Item** | **Description** | **Reported** | **Manuscript Section** | **Page No.** |
| --- | --- | --- | --- | --- | --- |
| 7 | Theoretical framework | Interpretivist paradigm used | Yes | Study design | 5 |
| 8 | Participant selection | Purposive sampling | Yes | Sampling and recruitment | 6 |
| 9 | Method of approach | Recruitment via hospital records | Yes | Sampling and recruitment | 6 |
| 10 | Sample size | 24 service users, 10 mental health workers | Yes | Sampling | 6 |
| 11 | Non-participation | Not reported | No | Not applicable | Not applicable |
| 12 | Setting of data collection | Private rooms in health facilities | Yes | Data collection procedures | 8 |
| 13 | Presence of non-participants | None present | Yes | Data collection procedures | 7 |
| 14 | Description of sample | Socio-demographic tables provided | Yes | Results (Tables 1-2) | 10-11 |
| 15 | Interview guide | Semi-structured guides used | Yes | Data collection procedures | 7 |
| 16 | Repeat interviews | Not conducted | No | Not applicable | Not applicable |
| 17 | Audio/visual recording | Audio recorded | Yes | Data collection procedures | 7 |
| 18 | Field notes | Field notes taken | Yes | Data collection procedures | 8 |
| 19 | Duration | 30-60 minutes per interview | Yes | Data collection procedures | 7 |
| 20 | Data saturation | Used as stopping criterion | Yes | Sampling and recruitment | 7 |
| 21 | Transcript returned | Member checking conducted | Yes | Trustworthiness | 8 |

**Domain 3: Analysis and findings**

| **No.** | **Item** | **Description** | **Reported** | **Manuscript Section** | **Page No.** |
| --- | --- | --- | --- | --- | --- |
| 22 | Number of data coders | Two independent coders | Yes | Data analysis | 8 |
| 23 | Description of coding tree | Themes and codes described | Yes | Results | 11-15 |
| 24 | Derivation of themes | Reflexive thematic analysis (inductive) | Yes | Data analysis | 6 |
| 25 | Software | NVivo used | Yes | Data analysis | 6 |
| 26 | Participant checking | Member checking conducted | Yes | Trustworthiness | 7 |
| 27 | Quotation presentation | Verbatim quotes included | Yes | Results | 11-15 |
| 28 | Data consistency | Triangulation used | Yes | Trustworthiness | 7 |
| 29 | Clarity of major themes | Five themes clearly defined | Yes | Results | 11-15 |
| 30 | Minor themes | Variations in emphasis reported | Yes | Cross-perspective triangulation | 15 |
| 31 | Clarity of findings | Well-structured thematic output | Yes | Results | 11-15 |
| 32 | Reflexivity | Researcher reflexivity acknowledged | Yes | Trustworthiness | 7 |
