# Supplementary material for: Treatment Experiences of Individuals With Co‐Occurring Mental Health and Substance Use Disorders and Perspectives of Mental Health Workers in Ashanti Region, Ghana
Source: Health Expect. 2026 Aug 3;29(4):e70801. doi: 10.1111/hex.70801 (PMC13430574; doi:10.1111/hex.70801)
Supplement: Supplementary file 2 — Supporting File 2 [file HEX-29-e70801-s001.docx]

**SUPPLEMENTARY FILE 2**

**Interview Guides**

**A. Participant Profiles**

| **Participant Group** | **Description** | **Recruitment Source** | **Eligibility Criteria** | **Interview Mode** | **Language** |
| --- | --- | --- | --- | --- | --- |
| Service Users (SU) | Individuals with co-occurring mental health and substance use disorders receiving or previously receiving care | Mampong Municipal Hospital, Adidwan Health Centre, Kofiase Health Centre | Adults (≥18 years), clinically stable, able to provide informed consent and reflect on experiences | Face-to-face, private clinical room | English / Twi (translation) |
| Mental Health Workers (MW) | Health professionals involved in assessment, treatment, or follow-up care of patients with co-occurring disorders | Same facilities (hospital and community outreach services) | Psychiatric nurses, general nurses in mental health units, allied health professionals with direct care experience | Face-to-face, private clinical room | English |

**B. Semi-Structured Interview Guide: Service Users (SU)**

**Introduction**

Participants will be welcomed and the purpose of the study explained in simple terms. Confidentiality, voluntary participation, and the right to withdraw at any time will be emphasized. Consent for audio recording will be obtained.

**Section 1: Understanding lived experience**

1. **Can you tell me about your journey with mental health and substance use?**

*Probes:* How did it begin? What changed in your life when it started?

1. **How would you describe your day-to-day life living with these challenges?**

*Probes:* Work, family, relationships, personal wellbeing

**Section 2: Experiences with treatment and care**

1. **Can you walk me through your experiences with receiving treatment in health facilities?**

*Probes:* First contact, ongoing care, changes over time

1. **What has your experience been like when trying to stay in treatment?**

*Probes:* What helps you continue? What makes it difficult?

1. **Can you describe any times you stopped or interrupted treatment? What influenced that?**

*Probes:* Cost, symptoms, distance, family issues, personal decisions

**Section 3: Substance use and recovery journey**

1. **How does substance use fit into your overall health experience?**

*Probes:* triggers, coping, relapse experiences

1. **What usually happens in your life when you relapse?**

*Probes:* emotional, social, financial consequences

**Section 4: Social environment and support**

1. **How have your relationships with family or community changed because of your condition?**

*Probes:* support, rejection, stigma

1. **How do people around you treat or respond to your situation?**

*Probes:* experiences of judgement, acceptance, discrimination

**Section 5: Financial and practical challenges**

1. **How have financial issues influenced your ability to access or continue treatment?**

*Probes:* transport, work, daily survival

**Section 6: Emotional experience and coping**

1. **How has this experience affected you emotionally or mentally?**

*Probes:* stress, hope, fear, motivation

**What helps you cope during difficult times?**

**Section 7: Health system experience and improvement**

1. **How would you describe your overall experience with mental health services?**

*Probes:* what works well, what does not

**If you could improve anything about the services, what would it be?**

**Closing question**

Is there anything about your experience that we have not discussed but you feel is important?

**C. Semi-Structured Interview Guide: Mental Health Workers (MW)**

**Introduction**

Purpose of study, confidentiality, and voluntary participation explained. Consent obtained before recording.

**Section 1: Professional experience**

1. **Can you describe your role in mental health care delivery?**

*Probes:* responsibilities, patient interaction, duration in service

1. **How would you describe your experience working with patients who have both mental health and substance use disorders?**

**Section 2: Clinical care and treatment pathways**

1. **How do patients with co-occurring disorders typically enter and move through the care system?**

*Probes:* referral pathways, continuity of care

1. **What challenges do you encounter in managing their treatment?**

*Probes:* relapse, adherence, follow-up

**Section 3: Health system and structural issues**

1. **What factors within the health system influence treatment outcomes for these patients?**

*Probes:* staffing, resources, coordination, workload

1. **How does service integration between mental health and substance use care function in practice?**
2. **How effective are community outreach and follow-up services in your experience?**

**Section 4: Social and patient-related factors**

1. **How do social and economic conditions of patients affect their treatment journey?**

*Probes:* transport, employment, family support

1. **What role does stigma play in care-seeking and adherence?**

**Section 5: Psychosocial care and emotional burden**

1. **How do you address the emotional and psychological needs of patients?**

*Probes:* counselling, limitations, frequency of support

1. **What challenges do you face in providing sustained psychosocial care?**

**Section 6: Improvements and recommendations**

1. **In** **your view, what changes are needed to improve care for these patients?**

*Probes:* policy, staffing, integration, community support

**Closing question**

Is there anything else you would like to add regarding care for patients with co-occurring disorders?
