# Supplementary material for: Treatment Experiences of Individuals With Co‐Occurring Mental Health and Substance Use Disorders and Perspectives of Mental Health Workers in Ashanti Region, Ghana
Source: Health Expect. 2026 Aug 3;29(4):e70801. doi: 10.1111/hex.70801 (PMC13430574; doi:10.1111/hex.70801)
Supplement: Supplementary file 3 — Supporting File 3 [file HEX-29-e70801-s003.docx]

**Coding Framework**

**Study:** Treatment experiences of individuals with co-occurring mental health and substance use disorders and perspectives of mental health workers in Mampong Municipality, Ghana

This codebook presents the final coding structure developed through reflexive thematic analysis. Codes were generated inductively from the data and iteratively refined into sub-themes and overarching themes. Representative quotes are provided to illustrate each code.

**Theme 1: Fragmented and unstable treatment pathways**

| **Sub-theme** | **Code** | **Code Definition** | **Example Quote** | **Data Source** |
| --- | --- | --- | --- | --- |
| Cyclical relapse-treatment pattern | Relapse-driven disengagement | Withdrawal from care triggered by relapse, disrupting continuity of treatment | “When I relapse, I come back worse than before…” (SU04) | Service users |
| Cyclical relapse-treatment pattern | Non-linear recovery trajectory | Recovery described as unstable, with repeated improvement and deterioration | “Sometimes I stop going. But when things become worse, I return again.” (SU11) | Service users |
| Service fragmentation | Inter-facility discontinuity | Movement between facilities disrupting continuity and requiring repeated reassessment | “Sent from Adidwan to Mampong… moving in circles.” (SU04) | Service users |
| Service fragmentation | Poor integration of dual diagnosis care | Lack of coordination between mental health and substance use treatment services | “Patients fall through the cracks.” (MW02) | Mental health workers |

**Theme 2: Hidden economic burden**

| **Sub-theme** | **Code** | **Code Definition** | **Example Quote** | **Data Source** |
| --- | --- | --- | --- | --- |
| Direct treatment costs | Transport-related financial barriers | Inability to attend treatment due to transport and indirect service costs | “I don’t have money for transport…” (SU09) | Service users |
| Employment instability | Loss of livelihood due to relapse | Job loss or interrupted vocational training due to relapse cycles | “Every relapse pushes me further away from learning the skill.” (SU15) | Service users |
| Coping strategies | Borrowing for treatment access | Reliance on informal loans or family support to access care | “Sometimes I borrow before I can come…” (SU09) | Service users |
| Structural poverty | Economic barriers to adherence | Poverty limiting treatment retention and reintegration | “Transport alone is a major barrier.” (MW06) | Mental health workers |

**Theme 3: Stigma, moral judgement, and social withdrawal**

| **Sub-theme** | **Code** | **Code Definition** | **Example Quote** | **Data Source** |
| --- | --- | --- | --- | --- |
| Community stigma | Social exclusion due to substance use history | Rejection or isolation from community due to drug use | “People think I am a bad person…” (SU02) | Service users |
| Internalised stigma | Self-stigma and shame | Internal feelings of worthlessness and guilt affecting behaviour | “I feel like people are watching me.” (SU02) | Service users |
| Anticipated stigma | Fear of judgement in healthcare settings | Avoidance of care due to expected negative attitudes | “I feel ashamed to go to the hospital.” (SU18) | Service users |
| Family concealment | Hidden illness management | Families hiding patients due to stigma | “Families sometimes hide patients.” (MW01) | Mental health workers |

**Theme 4: Emotional exhaustion and psychological distress**

| **Sub-theme** | **Code** | **Code Definition** | **Example Quote** | **Data Source** |
| --- | --- | --- | --- | --- |
| Psychological burden | Hopelessness and despair | Feelings of loss of hope regarding recovery | “I don’t want to continue anything.” (SU07) | Service users |
| Emotional instability | Fluctuating emotional states | Alternating emotional states linked to relapse cycles | “You feel better today and worse tomorrow.” (SU12) | Service users |
| Family-related guilt | Perceived burden on family | Emotional distress linked to family expectations | “I feel like I am disappointing my family.” (SU07) | Service users |
| Limited psychosocial support | Inadequate counseling support | Insufficient mental health support services for recovery | “Counseling is not enough.” (MW09) | Mental health workers |

**Theme 5: Health system constraints and follow-up gaps**

| **Sub-theme** | **Code** | **Code Definition** | **Example Quote** | **Data Source** |
| --- | --- | --- | --- | --- |
| Workforce limitations | Staff shortages and workload burden | Insufficient staff affecting care delivery and outreach | “Workload is high… staffing is limited.” (MW04) | Mental health workers |
| Outreach inconsistency | Irregular community follow-up | Inconsistent home visits affecting continuity of care | “It is not consistent.” (MW07) | Mental health workers |
| Delayed intervention | Late relapse response | Failure to detect relapse early due to weak monitoring systems | “Relapse before we reach them.” (MW07) | Mental health workers |
| Missed early care | Lack of early intervention | Patients deteriorate before follow-up occurs | “No early intervention.” (SU23) | Service users |
